# Histological study on the postnatal development of the nerve network in the rat ileal mucosa and submucosa

**DOI:** 10.1007/s00441-025-03949-3

**Published:** 2025-02-13

**Authors:** Rinako Morishita, Satoki Nakanishi, Toshifumi Yokoyama, Nobuhiko Hoshi, Youhei Mantani

**Affiliations:** 1 Laboratory of Histophysiology, Department of Bioresource Science, Graduate School of Agricultural Science, Kobe University, 1-1 Rokkodai-Cho, Nada-Ku, Kobe, Hyogo 657-8501 Japan; 2 Laboratory of Animal Molecular Morphology, Department of Bioresource Science, Graduate School of Agricultural Science, Kobe University, 1-1 Rokkodai-Cho, Nada-Ku, Kobe, Hyogo 657-8501 Japan

**Keywords:** Enteric nervous system, Mucosal nerve network, Postnatal development, Submucosal ganglion, Histological analysis

## Abstract

We have previously reported detailed structures of the mucosal nerve network in the rat ileum, but the mechanisms underlying the development of this nerve network remain unclear. Therefore, we aimed to clarify the developmental process of the mucosal nerve network and submucosal neurons (SM-neurons) or ganglia (SMG), which are the main source of nerve fibers projected to the mucosa, in the rat ileum during the postnatal period. Immunohistochemistry against tubulin beta III (Tuj1) revealed that Tuj1-immunopositivities were more abundant in the lamina propria at 2 weeks old (2wk; pre-weaning) than at postnatal day 0 (P0) or 4 weeks old (4wk; post-weaning) and more frequent on the mesenteric side than on the antimesenteric side at 2wk. Hu antigen D (HuD)-immunopositive SM-neurons and SMG were also more abundantly localized on the mesenteric side than the antimesenteric side at P0 and 2wk. On the other hand, cells immunopositive for SRY-related HMG-box 10 (Sox10), which is the marker for enteric nervous system progenitor cells and enteric glial cells, were homogenously scattered in the submucosa throughout the entire circumference at all ages. Glial cell marker S100 calcium-binding protein B (S100β) in the submucosa was detected at all ages without any significant difference between the mesenteric and antimesenteric sides. These findings indicate that SMG formation and associated neurite extension into the mucosa in the rat ileum might occur preferentially on the mesenteric side by the weaning period, leading us to hypothesize that the mechanism by which the mucosal nerve network and SMG develop differs along the mesenteric–antimesenteric side axis.

**Supplementary Information:**

The online version contains supplementary material available at 10.1007/s00441-025-03949-3.

## Introduction

A complex nervous network is developed within the intestinal mucosa of animals. This mucosal nerve network is derived from the enteric nervous system (ENS) or extrinsic nerve and plays an important role in the control of basic physiological functions in the intestinal mucosa. Physiological experiments have suggested that neurons innervating into the intestinal mucosa contribute to the regulation of mucosal motility and intestinal fluid secretion (Furness 2006). On the other hand, ultrastructural analysis with transmission electron microscope has shown that those mucosal nerve fibers are in contact with subepithelial fibroblasts or fibroblast-like cells (Desaki et al. 1984; Güldner et al. 1972; Nagahama et al. 2001) and mast cells (Newson et al. 1983; Stead et al. 1987). Furthermore, our previous studies using three-dimensional imaging technology with electron microscopy have shown that the nerve fibers of the intestinal mucosa contact numerous cell types, including fibroblast-like cells, macrophages, eosinophils, and plasma cells (Nakanishi et al. 2020, 2023). These findings collectively suggest that the mucosal nerve network plays diverse roles beyond the regulation of mucosal motility or secretion of intestinal fluid. Therefore, understanding the complex structure of the mucosal nerve network should be an important issue which provides novel insight into the significance of the ENS or extrinsic nerve in the intestinal mucosa. However, while the ultimate structure of the mucosal nerve network is increasingly being elucidated, the process and mechanisms underlying the development of this nervous network remain unclear.

The mechanisms by which ENS neurons extend their axons into the mucosa are still under investigation. Sonic hedgehog (Shh) is expressed in the epithelium in fetal mice, and Shh^−/−^ mice exhibit ectopic migration in the intestinal mucosa (Ramalho-Santos et al. 2000). Conditional knockout of Gas1, a receptor for Shh, in neural crest cells including ENS progenitor cells leads to excessive axon projection into the mucosa in E18.5 mice (Jin et al. 2015). Based on these findings, at least Shh secreted from the fetal epithelium is considered to have a role as a chemorepellent factor for enteric neurons, although the chemoattractive factors that induce axon projection into the mucosa are still under investigation. In any case, there can be no doubt regarding the importance of mucosal cells in the mechanisms underlying development of the mucosal nerve network. On the other hand, the structure or physiological status of the intestinal mucosa—including the epithelium—changes during the postnatal period in association with weaning (Cummins et al. 1988; Herbst and Sunshine 1969; Miller 1971; Vigueras et al. 1999). Therefore, we speculate that the mucosal nerve network might change during the postnatal period in tandem with the mucosal changes. However, this straightforward hypothesis regarding postnatal change in the mucosal nerve network remains unverified. In the present study, therefore, we first sought to elucidate the postnatal changes in the structure of the mucosal nerve network in the rat small intestine. In addition, in the process of histologically examining these changes, we found that the development of the mucosal nerve network proceeded preferentially on the mesenteric side of the small intestine. This unexpected finding led us to hypothesize that the submucosal ganglia (SMG), the major source of the mucosal nerve network (Keast et al. 1984), might develop on the mesenteric side preferentially. Therefore, our second aim in this study was to explore the difference in postnatal neuron and glial development in the SMG between the mesenteric and antimesenteric side.

## Methods

### Animals

Twelve pregnant female Wistar rats (gestation period 13–15 days) were purchased from Japan SLC (Hamamatsu, Japan) and housed separately. They were maintained under specific pathogen-free conditions in individual ventilated cages (Sealsafe Plus GR900; Tecniplast S.p.A, Buguggiate, Italy) with controlled temperature (23 ± 2 °C) and humidity (50 ± 10%) on a 12/12-h light/dark cycle at the Kobe University Life-Science Laboratory. All animals were permitted free access to water and food (Lab RA-2; Nosan Corp., Yokohama, Japan). Pups were separated from their mothers at 3 weeks of age, so 2-week-old rats were defined as pre-weaning rats, and 4-week-old rats were defined as post-weaning rats in this study. Thirty-seven male pups were used for this study at the ages of 0 days (P0, *n* = 14), 2 weeks (2wk, *n* = 11) and 4 weeks (4wk, *n* = 12). This experiment was approved by the Institutional Animal Care and Use Committee (permission numbers 30–05–01 and 2023–04–01) and carried out according to the Kobe University Animal Experimentation Regulations.

### Tissue preparation for light microscopy

Animals were anesthetized in accordance with the *Statement about sedation, anesthesia, and euthanasia in a rodent fetus and newborn* (second edition, 2015) from the Japanese College of Laboratory Animal Medicine; P0 rats were deeply anesthetized by placing them in a Falcon tube containing isoflurane (FUJIFILM Wako Pure Chemical Corp., Osaka, Japan) and cooling it on ice, and then decapitated; 2wk and 4wk rats were anesthetized by inhalation of an overdose of isoflurane. After euthanasia, small pieces were removed from the terminal region of the ilea (the region closest to the ileocecal junction). Then, each tissue block was immersion-fixed in 4.0% paraformaldehyde fixative in phosphate buffer for 6 h at 4 °C and snap-frozen in liquid nitrogen as described previously (Mantani et al. 2018). Sections were cut at a thickness of 4-µm using a Cryostat Leica CM1950 (Leica Biosystems, Nußloch, Germany), placed on slide glasses precoated with 0.2% 3-aminopropyltriethoxysilane (Shin-Etsu Chemical Co., Tokyo), and stored at − 30 °C until use.

### Immunohistochemistry

Antigens were detected using the modified indirect method of immunohistochemistry with the antibodies described below. Immunohistochemistry was performed using pan-neuronal markers (tubulin beta III (Tuj1) and Hu antigen D (HuD)), a marker for neural crest-derived cells and glial cells (SRY-related HMG-box 10 (Sox10)) or either of two markers exclusively for glial cells (glial fibrillary acidic protein (GFAP) and S100 calcium-binding protein B (S100β)). Briefly, the tissue sections were rinsed three times in 0.05% Tween-added 0.01 M phosphate-buffered saline (TPBS) after each preparation step to remove any reagent residues. All sections were then immersed in absolute methanol and 1.2% H_2_O_2_. Following blocking with Blocking One Histo (Nacalai Tesque, Kyoto, Japan) for 1 h at r.t., the sections to be used for the enzyme immunohistochemical analysis were reacted with anti-human Tuj1 rabbit polyclonal IgG (diluted 1:40,000, Cat# ab18207, RRID: AB_444319; Abcam, Cambridge, UK), anti-human S100β rabbit monoclonal IgG (diluted 1:400, Cat# ab52642, RRID: AB_882426; Abcam), or anti-porcine GFAP mouse monoclonal IgG (diluted 1:400, Cat# NBP1-05197SS, RRID: AB_11002767; Novus Biologicals, Littleton, CO, USA) for 18 h at 6 °C. The sections to be used for immunofluorescence analysis were reacted for 18 h at 6 °C with either of two pairs of antibodies: anti-mouse HuD rabbit polyclonal IgG (diluted 1:3,200, Cat# GTX134099, RRID: AB_2887214; GeneTex, Irvine, CA, USA)/anti-rat Tuj1 mouse monoclonal IgG (diluted 1:6,400, Cat# MAB1195, RRID: AB_357520; R&D Systems, Minneapolis, MN, USA) or anti-human Sox10 rabbit monoclonal IgG (diluted 1:400, Cat# NBP2-67,812; RRID: AB_3086734; Novus Biologicals)/anti-human α-smooth muscle actin (α-SMA) mouse monoclonal IgG (diluted 1:400, Cat# ab7817, RRID: AB_262054; Abcam). Next, the tissue sections for the enzyme immunohistochemical analysis were incubated with horseradish peroxidase (HRP)-conjugated anti-rabbit IgG donkey IgG (diluted 1:200, Cat# 711–035–152, RRID: AB_10015282; Jackson ImmunoResearch, West Grove, PA, USA) or HRP-conjugated anti-mouse IgG donkey IgG (diluted 1:200, Cat# 715–035–151, RRID: AB_2340771; Jackson ImmunoResearch) for 1 h at r.t. Sections were rinsed three times in TPBS and three times in Tris buffer (pH 7.6), and then stained in 0.02% 3,3′-diaminobenzidine (Dojindo Lab., Mashiki, Japan) in Tris buffer with 0.0051% H_2_O_2_ and counterstained with hematoxylin. For double immunofluorescence, the tissue sections were reacted with Rhodamine Red™-X-conjugated anti-rabbit IgG donkey IgG (diluted 1:200, Cat# 711–295-152, RRID: AB_2340613; Jackson ImmunoResearch), Alexa Fluor 488-conjugated anti-mouse IgG rat IgG (diluted 1:200, Cat# 415–545-166, RRID: AB_2340283; Jackson ImmunoResearch) and DAPI (diluted at 1:1,000; Dojindo Lab.) for 1 h at r.t. Control sections were incubated with TPBS or non-immunized mouse or rabbit IgG instead of the primary antibody, and the results were used to discriminate the specific reactions for each immunohistochemical analysis. Sections for the enzyme immunohistochemical analysis were then observed under a light microscope, while those for immunofluorescence analysis were observed under a fluorescent microscope. Observation of each target was conducted using the sections from five or more rats.

### Histological measurement

Entire images of transverse tissue sections were captured at × 400 magnification for P0 and 2wk, and at × 200 magnification for 4wk, and each image dataset was aligned using the TrackEM2 plugin of the Fiji software package (Version 1.3.10, Schindelin et al. 2012, RRID: SCR_002285). Immunopositivity for Tuj1 or S100β was extracted using the color deconvolution function of the Image J 1.54 h software package (Version 3.0.3, RRID: SCR_003070). After setting the maximum of threshold sufficiently low to eliminate the faint background immunopositivity in the lamina propria or submucosa in the negative control tissue sections staining with rabbit normal IgG, the areas immunopositive for Tuj1 or S100β were calculated using this threshold. The frequency of Tuj1-immunopositivity was calculated by dividing the Tuj1-immunopositive area by the area of the lamina propria (Supplementary Fig. 1). The number of HuD^+^ or Sox10^+^ cells was determined using the multi-point tool in ImageJ. An SMG was defined as a cluster of two or more neurons, and the number of SMG was also determined. At 2wk and 4wk, the submucosa was identified as the layer between the *muscularis mucosae* and circular muscle layer. At P0, the submucosa was defined as the region between the line connecting the base of the intestinal crypts and the luminal line of the circular muscle layer because the *muscularis mucosae* were not clearly formed at this stage.

### Statistical analysis

Statistical analyzes were performed with BellCurve for Excel (Version 4.04; SSRI, Tokyo, Japan). For parametric variables in the comparison between the two groups with correspondence, a paired-samples *t*-test was performed. For nonparametric variables in the comparison between the two groups with correspondence, a Wilcoxon signed-rank test was performed. For nonparametric variables in multiple comparisons, the Kruskal − Wallis test was performed, followed by the Steel − Dwass test for post hoc comparisons. *P* values less than 0.05 were considered statistically significant.

## Results

### Postnatal change of Tuj1-immunopositivity in the lamina propria of the rat ileal mucosa

**Fig. 1:**
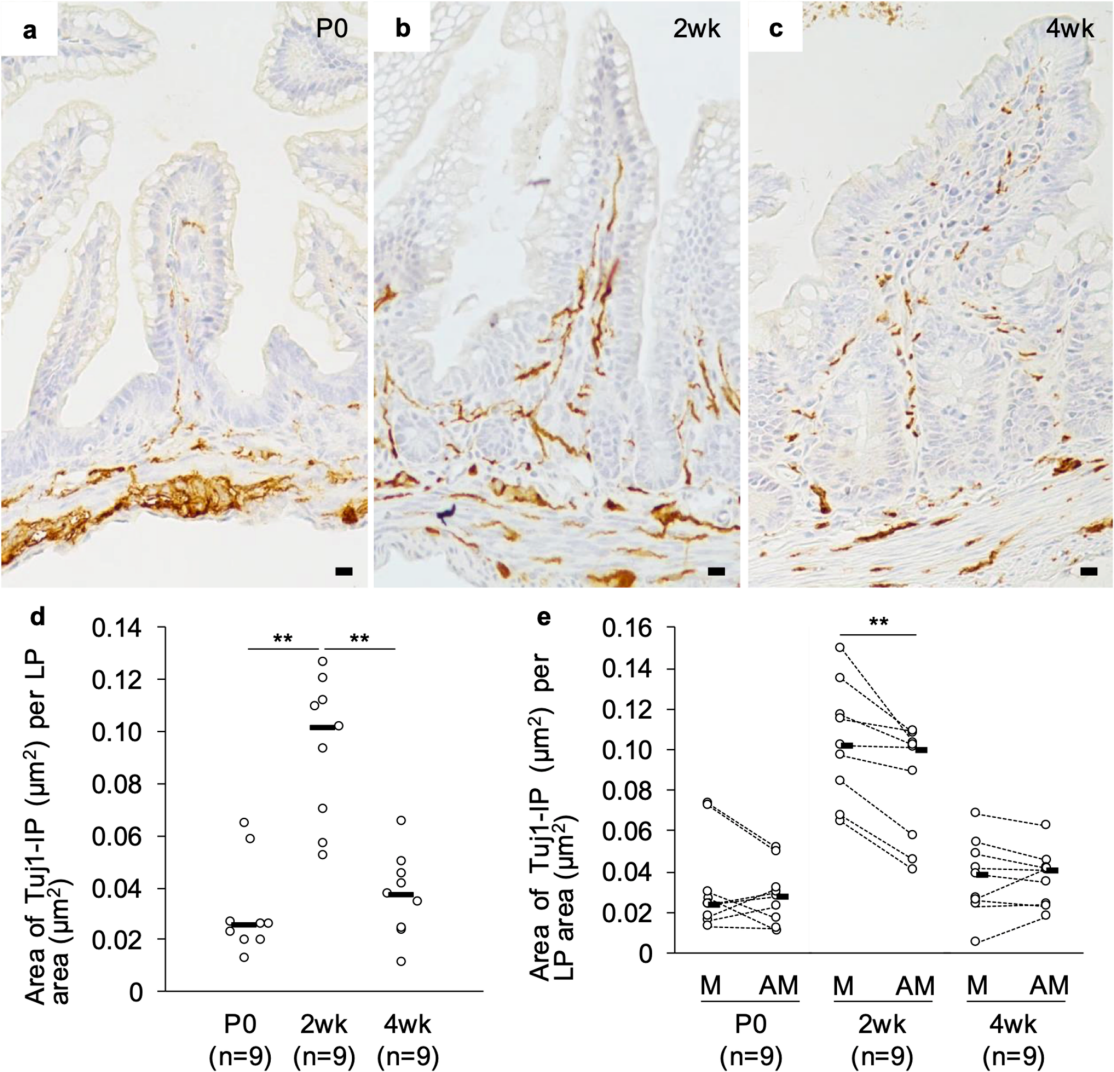
Postnatal change of the Tuj1^+^ nerve network in the lamina propria (LP) of the rat ileum. **a**–**c** Fibrous immunopositivity for Tuj1 was observed in the ileal LP of a postnatal day 0 (P0) rat (**a**), a 2-week-old (2wk) rat (**b**), and a 4-week-old (4wk) rat (**c**). Bars = 10 µm. **d** The area of Tuj1-immunopositivity (Tuj1-IP) per 1 µm^2^ area of LP in the rat ileum. Each median value is represented by a horizontal bar. Comparisons were performed by the Kruskal − Wallis test followed by the Steel − Dwass test. Double Asterisks, *P* < 0.01. **e** The area of Tuj1-IP per 1 µm^2^ area of LP on the mesenteric (M) or antimesenteric (AM) side of the rat ileum. Each median value is represented by a horizontal bar. Comparisons were performed by a Wilcoxon signed-rank test. Double asterisks, *P* < 0.01. The numbers in parenthesis are the sample sizes (**d, e**)

First, immunohistochemistry for the pan-neuronal marker Tuj1 was performed to investigate how the mucosal nerve network is altered during the postnatal period. Immunopositivity against Tuj1 was detected in the ileal lamina propria at all ages (Fig. 1a–c). Compared to that at P0 (Fig. 1a) or 4wk (Fig. 1c), Tuj1-immunopositivity was more frequent in the lamina propria at 2wk (Fig. 1b), which was supported by the histological measurement (Fig. 1d). Interestingly, the mucosal nerve network seemed to be more intricate on the mesenteric side than on the antimesenteric side, especially in 2wk. To further investigate this phenomenon, we divided the transverse sections of the ileum into mesenteric and antimesenteric sides and remeasured the Tuj1-immunopositivity in the lamina propria on both sides. The quantitative analysis showed that the frequency of Tuj1-immunopositivity in the lamina propria was not significantly different between the mesenteric side and antimesenteric side of transverse sections at P0 and 4wk, but significantly higher on the mesenteric side than on the antimesenteric side at 2wk (Fig. 1e). These findings suggest that the mucosal nerve network might be preferentially formed on the mesenteric side during the period from birth to weaning.

### Neurons and ganglia in the rat ileal submucosa increase preferentially on the mesenteric side during the postnatal period

**Fig. 2:**
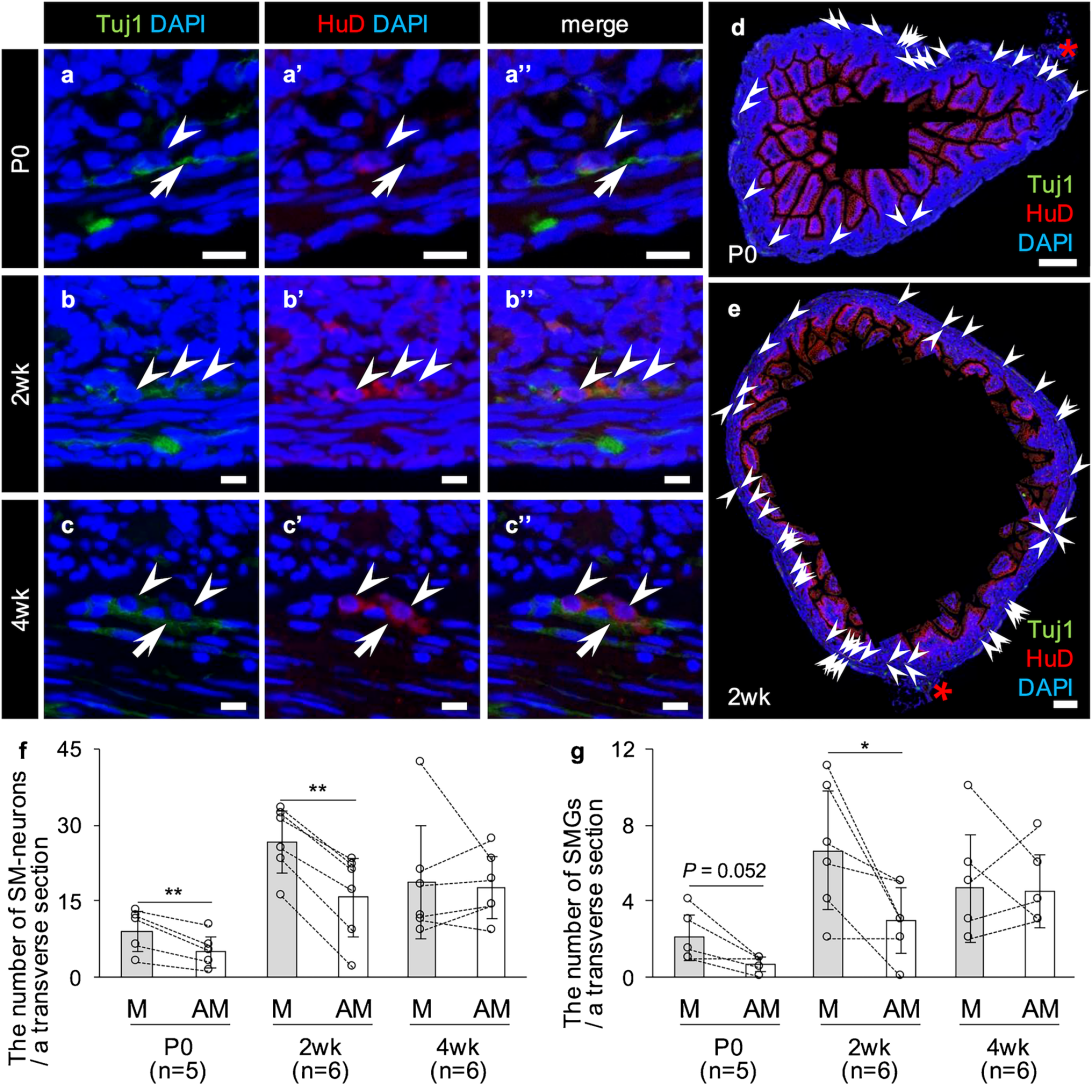
Postnatal change of HuD^+^ submucosal neurons (SM-neurons) and submucosal ganglia (SMG) of the rat ileum. **a–c** Immunofluorescence against Tuj1 (green)/HuD (red). Immunopositivities for HuD (white arrowheads) are observed in the cellular bodies of neurons in the submucosa of the ileum in a postnatal day 0 (P0) rat (**a**), a 2-week-old (2wk) rat (**b**), and a 4-week-old (4wk) rat (**c**), whereas those for Tuj1 are observed in their nerve fibers (white arrows). Bars = 10 µm. **d–e** Whole view of transverse sections with immunofluorescence against HuD (red)/Tuj1 (green) from a P0 (**d**) and a 2wk (**e**) rat. High magnification images of individual HuD^+^ SM-neurons are shown in Supplementary Fig. 2. Bars = 100 µm. Arrowheads, HuD^+^ SM-neurons. Red asterisks, mesentery. **f** The number of SM-neurons per a transverse section on the mesenteric (M) or antimesenteric (AM) side of the rat ileum. Results are shown as the means ± SD. Comparisons were performed by a paired-sample *t*-test. Double asterisks, *P* < 0.01. **g** The number of SMG per a transverse section on the M or AM side of the rat ileum. Results are shown as the means ± SD. Comparisons were performed by a paired-sample *t*-test. Asterisk, *P* < 0.05. The numbers in parenthesis are the sample sizes (**f, g**)

The above finding that the ileal mucosal nerve network is preferentially formed on mesenteric side led us to formulate the novel hypothesis that mesenteric side-preferential postnatal changes might also occur in the SMG, whose neurons are the main source of nerve fibers projecting to the mucosa. Next, therefore, we sought to analyze postnatal changes in submucosal neurons (SM-neurons) and SMG. Double immunofluorescence for two pan-neuronal markers, i.e., Tuj1 and HuD, revealed that HuD was present in the cytoplasm of neurons (Fig. 2a–c), although it was also detected on the lateral surface of epithelial cells. On the other hand, immunopositivities for Tuj1 were mainly detected in the portions of nerve fibers (Fig. 2a–c), making it difficult to count the number of neurons. Therefore, HuD was adopted as the neuronal marker for histological analysis of SM-neurons and SMG in this study. SM-neurons and SMG were few in P0 (Fig. 2a), and more abundant at 2wk and 4wk than at P0 (Fig. 2b, c). Interestingly, HuD-immunopositive cells were significantly more abundant on the mesenteric side at P0 and 2wk (Fig. 2d–f, Supplementary Fig. 2). On the other hand, there was no clear difference between the two sides at 4wk (Fig. 2f). Similarly, the number of SMG was significantly higher on the mesenteric side than on the antimesenteric side at 2wk (*P* < 0.05) (Fig. 2g).

### Postnatal change in cellular markers for ENS-progenitor cells or glial cells in the rat ileal submucosa

**Fig. 3:**
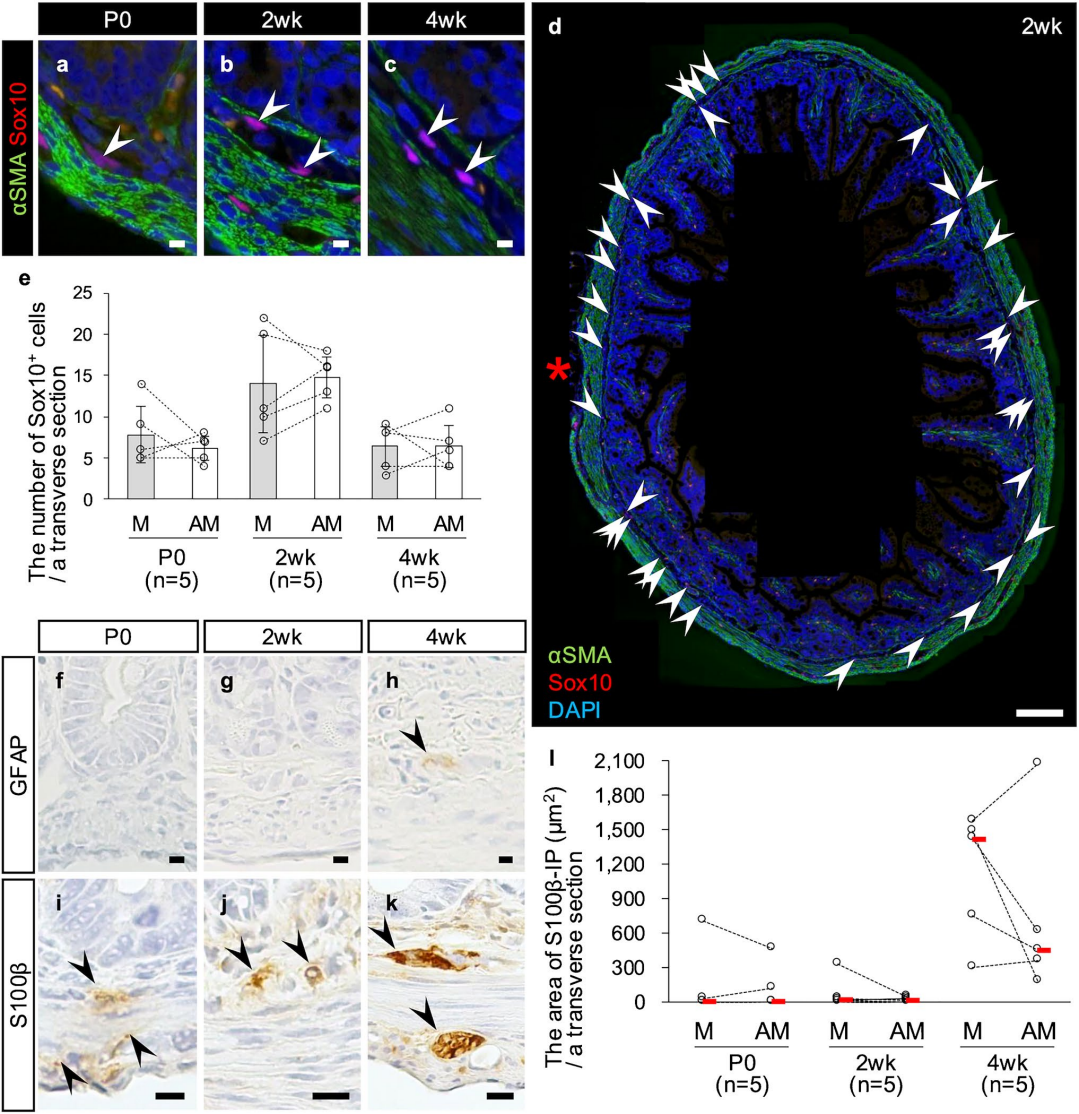
Postnatal change in the expressions of Sox10, GFAP and S100β in the submucosa of the rat ileum. **a–c** Immunofluorescence against Sox10 (red)/α-SMA (green, marker for smooth muscle cells). Oval nuclei immunopositive for Sox10 (white arrowheads) are observed in the ileal submucosa of a postnatal day 0 (P0) rat (**a**), a 2-week-old (2wk) rat (**b**), and a 4-week-old (4wk) rat (**c**). **d** Whole view of a transverse section with immunofluorescence against Sox10 (red)/α-SMA (green) from a 2wk rat. Arrowheads, Sox10^+^ cells in the submucosa. Red asterisk, mesentery. **e** The number of Sox10^+^ cells in the submucosa per a transverse section on the mesenteric (M) or antimesenteric (AM) side of the rat ileum. Results are shown as the means ± SD. Comparisons were performed by a paired-sample *t*-test. **f–h** Immunohistochemistry for GFAP in the rat ileal submucosa at P0 (**f**), 2wk (**g**), and 4wk (**h**). Immunopositivity for GFAP in the submucosa is not observed at P0 (**f**) or 2wk (**g**) but is weakly observed at 4wk (**h**, arrowhead). **i–k** Immunohistochemistry for S100β in the rat ileal submucosa at P0 (**i**), 2wk (**j**), and 4wk (**k**). Immunopositivity for S100β in the ileal submucosa is observed at all stages, although it is weak at P0 and 2wk. Arrowheads, S100β^+^ cells. Bars = 10 µm (**a–c, f–k**) or 100 µm (**d**). **l** The area immunopositive for S100β (S100β-IP) per a transverse section on the M or AM side of the rat ileum. Each median value is represented by a red horizontal bar. Comparisons were performed by a Wilcoxon signed-rank test. The numbers in parenthesis are the sample sizes (**e**, **l**)

Neurons in the small intestine are derived from ENS-progenitor cells migrating from the vagal neural crest during fetal life (Le Douarin and Teillet 1973; Yntema and Hammond 1954). Therefore, we next examined the localization of ENS-progenitor cells in the submucosa to determine whether the preferential development of SM-neurons and SMG on the mesenteric side is attributable to a localization of ENS-neuronal progenitor cells. Immunohistochemistry against Sox10, which is a marker for ENS-progenitor cells and glial cells (Kim et al. 2003; Kuhlbrodt et al. 1998; Paratore et al. 2002), revealed that Sox10-immunopositive cells were clearly present in the submucosa at all ages, including at P0, and were more abundant at 2wk (Fig. 3a–c). Also at all ages, Sox10-immunopositive cells in the submucosa were homogenously scattered throughout the entire circumference, and there was no significant difference in the number of Sox10^+^ cells between the mesenteric and antimesenteric sides at any of the ages examined (Fig. 3d–e). Next, since Sox10 is also expressed in enteric glial cells (Kuhlbrodt et al. 1998), we examined postnatal changes in glial cells by immunohistochemistry against GFAP and S100β, two other markers for enteric glial cells (Boesmans et al. 2015; Ferri et al. 1982; Jessen and Mirsky 1983). Immunopositivity for GFAP was absent in the submucosa at both P0 and 2wk, but detected at 4wk with weak immunopositive intensity (Fig. 3f–h). S100β immunopositivity in the submucosa was weak in almost all individuals at P0 and 2wk, but was clearly seen at 4wk (Fig. 3i–k). The S100β-immunopositive area per a transverse section did not differ significantly between the mesenteric and antimesenteric sides at any of the ages examined (Fig. 3l). These results suggest that Sox10-immunopositive cells at all ages and S100β-immunopositive cells at 4wk are evenly distributed over the entire circumference, and that S100β^+^ glial cells increase later than neurons at around 4wk after weaning.

## Discussion

In the rat small intestine, the structure and function of the mucosa undergo major changes during the transition from suckling to weaning. For example, in the neonatal rat ileum, the size and number of intestinal villi and the depth of the intestinal crypts increase dramatically from birth to 35 days of age (Herbst and Sunshine 1969), with a slower rate of increase thereafter (Vigueras et al. 1999). Moreover, the number of mucosal cells, such as goblet cells, eosinophils, intraepithelial lymphocytes and mucosal mast cells, per unit length in the jejunum of 24-day-old rats is 3–5 fold higher than that of 12-day-old rats (Cummins et al. 1988). In the present study, Tuj1-immunopositivity in the rat ileal lamina propria was more abundant at 2wk than at P0. This result in the lamina propria was consistent with the observation that neurons and ganglia in the submucosa were more abundant at 2wk than at P0. These results suggest that the nerve network structure in the rat ileal mucosa is not fully formed at birth, and that an increasing number of SM-neurons actively extend nerve fibers into the mucosa over the period up to weaning to form the mature mucosal nerve network structure necessary for the regulation of intestinal physiological functions after weaning.

In this study, we newly found that HuD-immunopositive SM-neurons are more abundant on the mesenteric side than on the antimesenteric side at P0 and 2wk. In contrast, cells immunopositive for Sox10, which is expressed in ENS-progenitor cells (Kim et al. 2003; Paratore et al. 2002) and enteric glial cells (Kuhlbrodt et al. 1998), were uniformly scattered throughout the entire circumference of the submucosa at all stages examined. On the other hand, immunohistochemistry against the other enteric glial cell markers, GFAP and S100β (Boesmans et al. 2015; Ferri et al. 1982; Jessen and Mirsky 1983), revealed that immunopositivity for S100β, but not GFAP, was present at all ages without preference toward the mesenteric side. These findings indicate that ENS-progenitor cells are uniformly distributed throughout the entire circumference of the submucosa in P0 and 2wk rats, which in turn suggests that the preference for SM-neuron localization to the mesenteric side might be attributed to active differentiation into neurons on the mesenteric side, rather than to the distribution of ENS-progenitor cells. In addition, given that SMG are formed by a subpopulation of cells that migrate radially from the myenteric layer to the submucosa (Kapur et al. 1992; Uesaka et al. 2013), we could not exclude the possibility that the postnatal migration of neurons from the myenteric ganglia to the submucosa occurs preferentially on the mesenteric side. These hypotheses should be examined in the future. On the other hand, glial cell marker S100β-immunopositivity in the submucosa was more abundant at the postweaning time point of 4wk than at P0 or 2wk. This finding suggests that glial cells in the submucosa, unlike neurons, might be increased by stimulation of the luminal environment associated with weaning.

Although the mechanism underlying the preferential increase in SM-neurons or SMG formation on the mesenteric side remains speculative, extrinsic nerves entering the intestine from the mesentery-attachment site might contribute to its mechanism. For example, Schwann cell precursors (SCPs) have been shown to enter the small intestinal wall via extrinsic nerves at approximately E14.5 and differentiate into SM-neurons at least until 1 month old (Uesaka et al. 2015). This suggests that mesenteric side-preferential development of SM-neurons observed in this study might be partly attributed to SCPs entering from the mesenteric side. However, considering that SCP-derived neurons account for only approximately 5% of the total SM-neurons in the small intestine in 1-month-old mouse (Uesaka et al. 2015), there must be other factors that contribute to the mesenteric side-preferential development of SM-neurons as well. In addition to extrinsic nerves, blood vessels might be one of the other contributors as they also enter the intestinal wall at the mesentery attachment site. Indeed, blood vessels have been reported to serve as scaffolds for neuronal migration in both the central nervous system and ENS (reviewed in Fujioka et al. 2019). For instance, in the intestine of quail embryos, two concentric blood vessels develop before ENS development, and inhibition of vascular development with a vascular endothelial growth factor receptor inhibitor leads to aganglionosis in the distal intestine, suggesting that blood vessels are required for the migration of ENS-progenitor cells (Nagy et al. 2009). Based on these findings, blood vessels entering from the mesenteric attachment site might also contribute to the preferential migration of neurons or ENS-progenitor cells to the submucosa on the mesenteric side discussed above.

Based on the present findings, we hypothesized that the formation of the mucosal nerve network and SMG in the postnatal rat ileum progresses preferentially on the mesenteric side, as discussed above. However, there are still some unresolved issues. Interestingly, the frequency of Tuj1-immunopositivity in the rat ileal mucosa decreased from 2 to 4wk, but at present, we could not decipher the mechanism underlying this finding. The frequency of Tuj1-immunpositivity measured in this study was calculated by dividing the Tuj1-immunopositive area in the lamina propria by the area of the whole lamina propria. Given this methodology, we can think of two potential explanations for the decrease in the frequency of Tuj1-immunopositivity in the mucosa from 2 to 4wk: (1) the area of the lamina propria may have increased without additional extension of nerve fibers and (2) the amount of nerve fibers may have decreased in the lamina propria. Indeed, excessively elongated nerve fibers are selected after birth according to the intensity of subsequent neural activity in the retina, cerebellum, cortex, and neuromuscular junction (reviewed in Faust et al. 2021); this phenomenon is called pruning (elimination). There is also a report suggesting that neurons are phagocytosed by macrophages in the early postnatal period in the myenteric ganglia of the mouse ileum (Viola et al. 2023). Based on the above reports, it is possible that the frequency of Tuj1-immunpositivity in the rat ileal mucosa decreased around the weaning period by pruning (elimination) of nerve fibers or neurons. Further study will be needed to examine this fascinating hypothesis in the future.

**Fig. 4:**
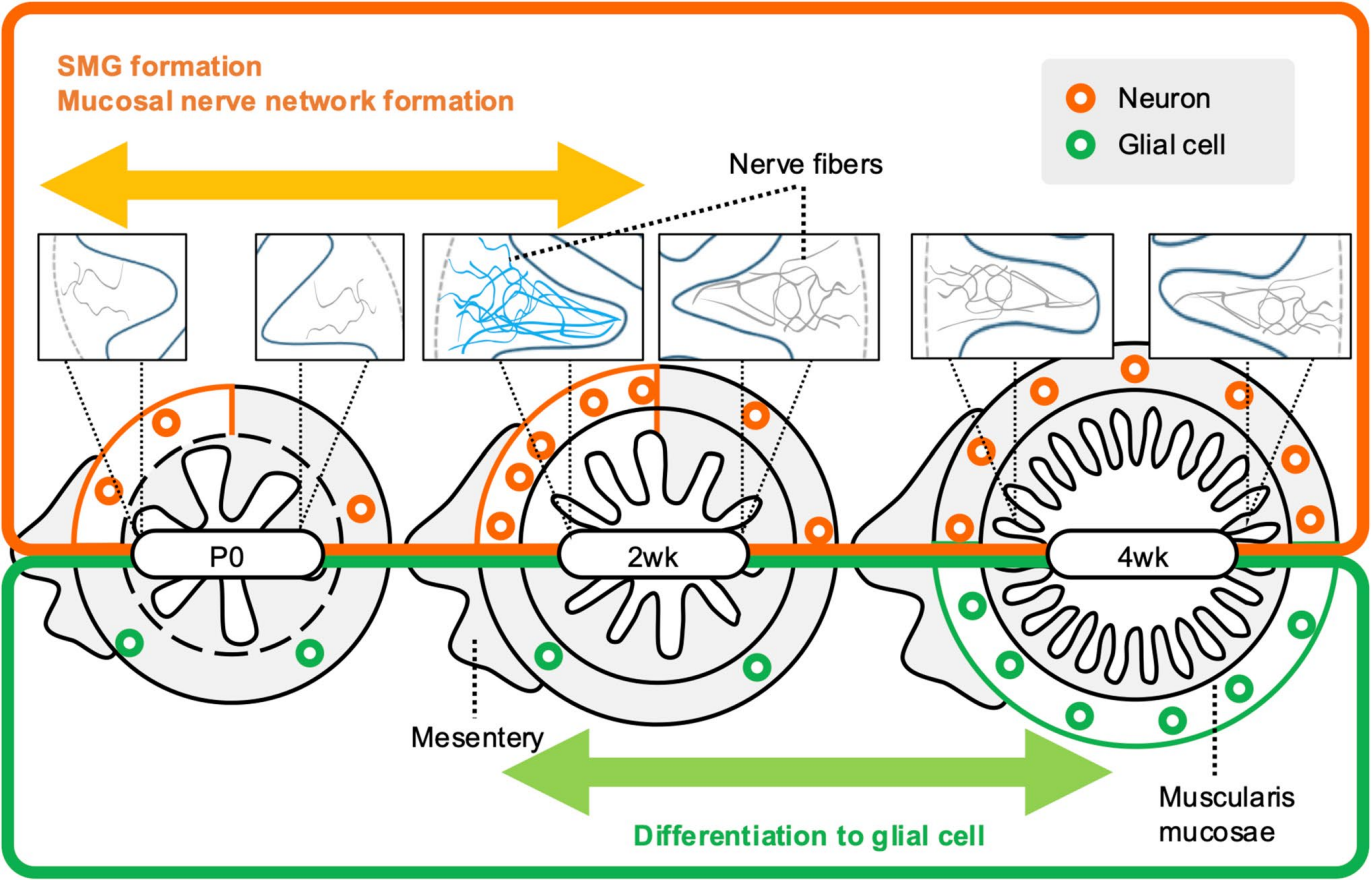
Schematic diagram of postnatal changes on the mucosal nerve network and submucosal neurons and glial cells in the rat ileum. P0, postnatal day 0. 2wk, 2 weeks of age. 4wk, 4 weeks of age

In conclusion, the present study clarified that the differentiation/migration of SM-neurons, SMG formation, and subsequent extension of neurites into the mucosa in the rat ileum might not occur uniformly throughout the entire circumference of the submucosa after birth, but rather may occur preferentially on the mesenteric side (Fig. 4). These findings lead us to propose a new hypothesis in regard to ENS development—namely, that the mechanism by which SMG develops differs along the mesenteric–antimesenteric side axis.

## Supplementary Information

Below is the link to the electronic supplementary material.

Supplementary file1 (PDF 555 KB)

## Data Availability

No datasets were generated or analysed during the current study.
