# Supplementary material for: Histological study on the postnatal development of the nerve network in the rat ileal mucosa and submucosa
Source: Cell Tissue Res. 2025 Feb 13;400(1):71–80. doi: 10.1007/s00441-025-03949-3 (PMC11965212; doi:10.1007/s00441-025-03949-3)
Supplement: Supplementary file 1 — Supplementary file1 (PDF 555 KB) [file 441_2025_3949_MOESM1_ESM.pdf]

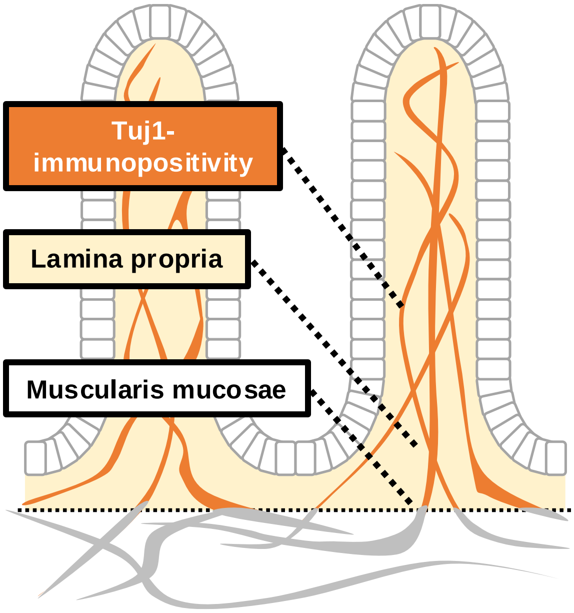

Supplementary Fig. 1: Schematic diagram of portions analyzed in the histological measurement of the area immunopositive for Tuj1 in the present study. The region of interest (ROI) was set to surround the lamina propria (LP) by using Image J software. After setting the ROI, the ratio of the Tuj1-immunopositive area to the total LP-area was calculated as the frequency of Tuj1-immunopositivity

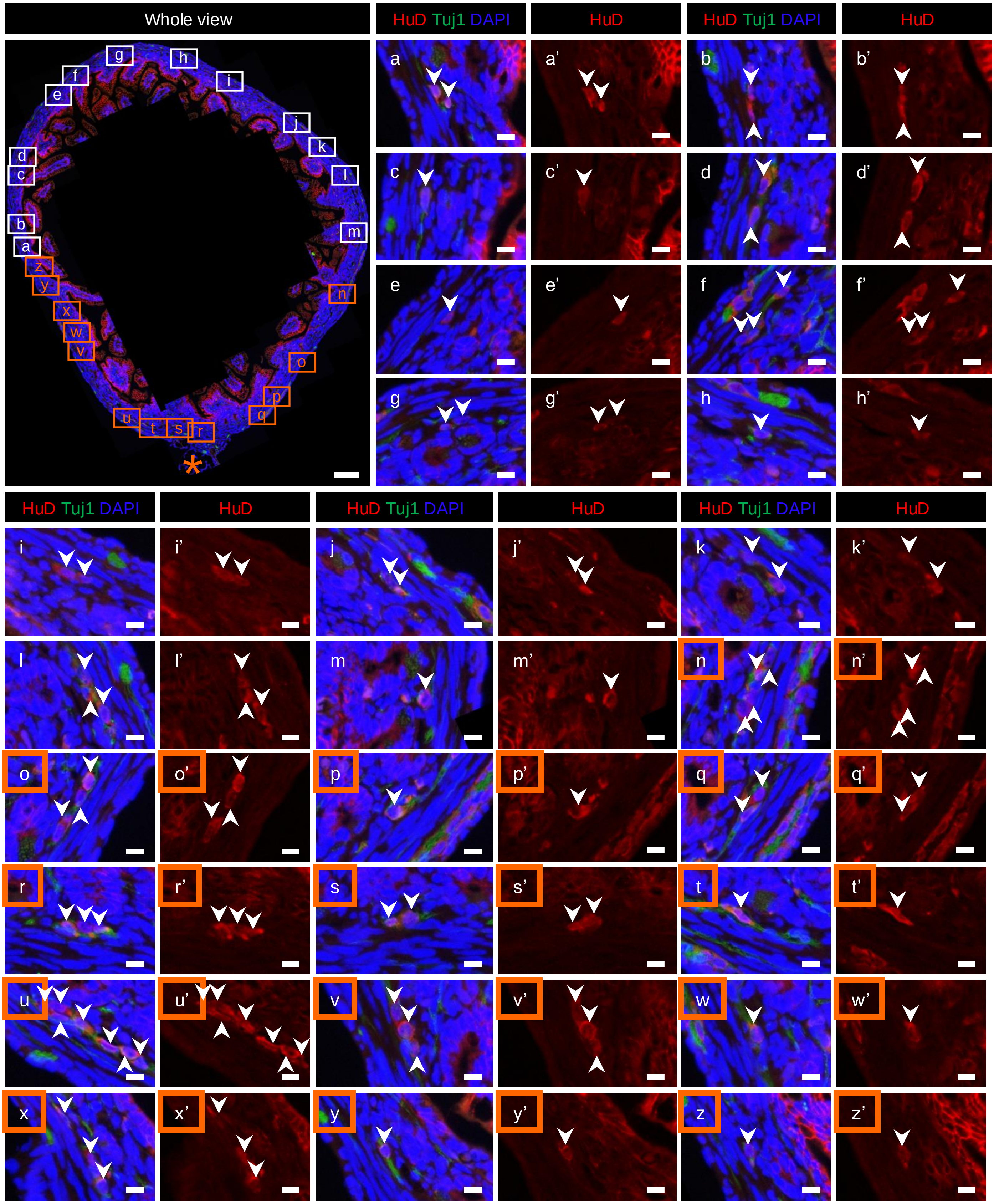

Supplementary Fig. 2: Whole view of a section with immunofluorescence against HuD (red) or Tuj1 (green) of 2wk (left upper) and corresponding high magnification images (antimesenteric side: **a-m;** mesenteric side: **n-z**). HuD^+ submucosal neurons (white arrowheads) are located preferentially on the mesenteric side. Bars = 100 µm (whole view) or 10 µm (magnified view). Asterisk, mesentery
